# A novel mutation in *EYA1* in a Chinese family with Branchio-oto-renal syndrome

**DOI:** 10.1186/s12881-018-0653-2

**Published:** 2018-08-07

**Authors:** Yan-gong Wang, Shu-ping Sun, Yi-ling Qiu, Qing-he Xing, Wei Lu

**Affiliations:** 1 Institutes of Biomedical Sciences and Children’s Hospital, Fudan University, Mingdao Building, Dong-an Road 131, Shanghai, 200032 China; 2 Department of Otorhinolaryngology, The First Affiliated Hospital of Zhengzhou University, No. 1 Jian-she Road, Zhengzhou, 450052 China; 3 The Center for Pediatric Liver Diseases, Children’s Hospital of Fudan University, Wan-yuan Road 399, Shanghai, 201102 China

**Keywords:** Chinese family, Branchio-Oto-renal syndrome, *EYA1*, Mutation

## Abstract

**Background:**

Branchio-oto-renal (BOR) syndrome is a dominant autosomal disorder characterized by phenotypes such as hearing loss, branchial fistulae, preauricular pits, and renal abnormalities. *EYA1*, the human homolog of the *Drosophila* “eye absent” gene on chromosome 8q13.3, is recognized as one of the most important genes associated with BOR syndrome.

**Methods:**

The proposita in this study was a 5-year-old Chinese girl with hearing loss, bilateral otitis media with effusion, microtia, facial hypoplasia, palatoschisis, and bilateral branchial cleft fistulae. The girl’s family members, except two who were deceased, agreed to undergo clinical examination. We collected blood samples from 10 family members, including six who were affected by the syndrome. Genomic DNA was extracted and subjected to Sanger sequencing. A minigene assay was performed to confirm whether splicing signals were altered. In addition, we performed western blotting to determine alterations in protein levels of the wild-type and mutant gene.

**Results:**

Clinical tests showed that some of the family members met the criteria for BOR syndrome. The affected members harbored a novel heterozygous nonsense variation in exon 11 of *EYA1*, whereas no unaffected member carried the mutation at this position. Functional experiments did not detect abnormal splicing at the RNA level; however, western blotting showed that the mutated protein was truncated.

**Conclusions:**

This study reports a novel mutation associated with BOR syndrome in a Chinese family. We highlight the usefulness of genetic testing in the diagnosis of BOR syndrome. Thus, we believe that this report would benefit clinicians in this field.

**Electronic supplementary material:**

The online version of this article (10.1186/s12881-018-0653-2) contains supplementary material, which is available to authorized users.

## Background

Branchio-oto-renal (BOR) syndrome (OMIM113650) is a hereditary dominant autosomal disease with a variable spectrum of manifestations [1]. Currently, most physicians follow the clinical diagnostic criteria based on the principles of Chang [2] to diagnose BOR syndrome. Although scientists reported cases with ear, branchial, and kidney anomalies in the early nineteenth century, Melnick [3] and Fraser [4] were the first to describe these phenotypes in detail. The clinical manifestations of BOR syndrome are highly heterogeneous. Patients with BOR syndrome and ear and branchial defects can be easily identified in early childhood, whereas more time is required for identifying kidney defects [5, 6]. Some cases of kidney abnormalities are detected at infancy, whereas others are detected in adulthood, which delays the appropriate time for therapeutic intervention. This indicates that more attention should be paid to the diagnosis of BOR syndrome as early as possible.

In 1992, Smith and Kumar [7] used linkage studies to locate the pathogenic gene on chromosome 8q13.3. In 1997, scientists [8] studied seven patients with BOR syndrome and identified a novel causative gene, known as *EYA1* (eyes absent). Chang et al. [2] demonstrated that approximately 40% patients with BOR syndrome harbored *EYA1* mutations. *EYA1* is a conserved transcriptional co-activator [9] and is highly expressed in the human embryonic kidney, whereas it is scantily expressed in the brain and lung. In adults, *EYA1* is strongly expressed in the heart and skeletal muscles, whereas weaker expression has been observed in the liver and brain; no expression has been observed in the eyes and kidney [10]. In addition, *EYA1* is subject to the gene dosage effect, which implies that the quantity of the encoded protein decides the development of the branchial arch, ear, and kidney; the gene activity is discernible only if the encoded protein quantity surpasses a certain threshold. This can explain the differences in phenotypes among BOR patients in one family.

Although previous studies have reported many BOR-related mutations [11–16], this is the first time that BOR syndrome has been investigated genetically and pathogenic mutations in *EYA1* have been detected in a Chinese family with BOR syndrome.

## Methods

### Subjects

**Fig. 1:**
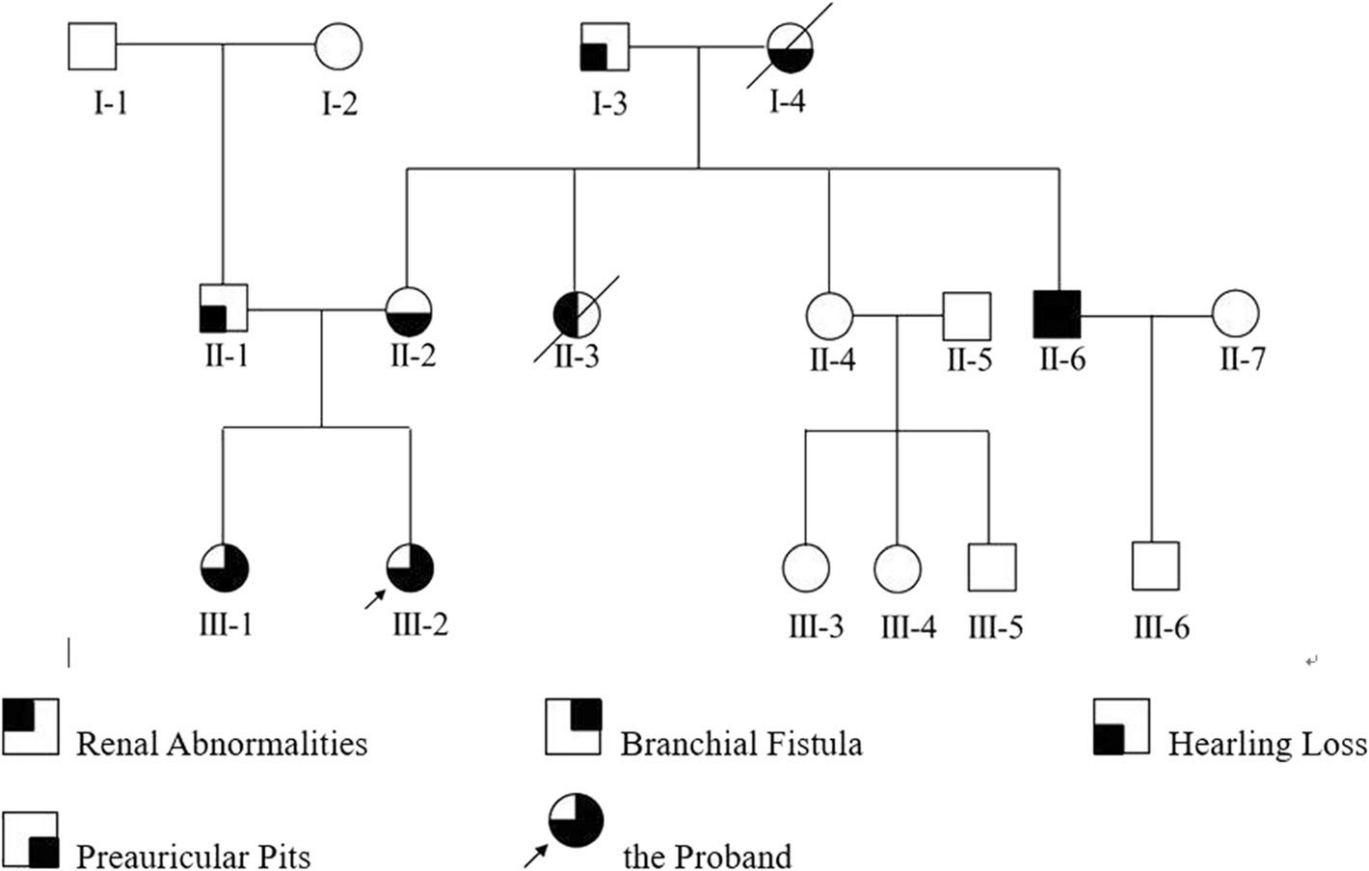
Pedigree of a family with branchio-oto-renal (BOR) syndrome

We collected the pedigree information of 17 members of a family over three generations at the First Affiliated Hospital of Zhengzhou University, China (Fig. 1).

Figure 1 shows the relationship and the prevalence situation of this pedigree with BOR syndrome. The four main features of BOR syndrome are identical to the criteria used for distinguishing disease progression (renal abnormalities, branchial fistula, hearing loss, and preauricular pits) [2]. The other family members of II:1 (I:1, I:2, II:4, II:5, II:7, III:3, III:4, III:5, and III:6) were normal.

### Sanger test

Venous blood was obtained for screening *EYA1* mutations from 10 living members (I:1, I:2, I:3, II:1, II:2, II:4, II:6, III:1, III:2, and III:6) of this family after obtaining informed consent. Genomic DNA was isolated using the AxyPrep blood genomic DNA miniprep kit (Axygen Biosciences, CA, USA) per manufacturer’s instructions. Eighteen primers corresponding to 18 exons of *EYA1* (NM_000503) were designed using the online tool Primer3web (http://primer3.ut.ee/) (Additional file 1: Table S1). The sequencing was performed using an ABI BigDye Terminator cycle sequencing kit (Applied Biosystems, CA, USA) on an ABI PRISM 3730 DNA Analyzer (Applied Biosystems), and the data were analyzed using the software CodonCode Aligner and Mutation Surveyor V4.0.5. SIFT, Polyphen2, and MutationTaster [17] were used to predict the pathogenicity of the variant. The mutation site was extensively analyzed using the online tool MutationTaster (http://www.mutationtaster.org/).

### Minigene experiment

The genomic DNA of III:1 (control sample) and III:2 (case sample) were PCR amplified with Q5 hot start high-fidelity DNA polymerases (New England Biolabs, MA, USA) for exon 11 of *EYA1* (2,051 bp total, 967 bp upstream, and 1,000 bp downstream, according to c.967A). Next, the PCR products were digested with *Xba*I and *Xho*I (New England Biolabs) and ligated in carrier pCMV-Tag-2B (Agilent Technologies) to generate pCMV-con (c.967A) and pCMV-case (only c.967 T was picked). The two plasmids were transformed in 293 T cells using Lipofectamine 2000 (Invitrogen Life Technologies, Carlsbad, CA, USA) following the manufacturer’s instructions. After 12 h, the medium was changed to Dulbecco’s modified Eagle medium plus 10% fetal bovine serum (Gibco) and incubated for another 24 h till the total RNA levels reached the maxima. Then, the two plates of cells (pCMV-con and pCMV-case) were treated with Trizol, and RNA samples were obtained. We sequenced the two cDNAs synthesized from the extracted RNA using the PrimeScript™ RT reagent kit (Takara, Osaka, Japan).

### Western blot analysis

The cDNA of *EYA1* was inserted into pRK7-N-Flag between *Xba*I and *Eco*RI to construct the vector pRK7-conl. c.967A was mutated to c.967 T in the pRK7-conl vector to obtain pRK7-case using the Takara MutanBEST kit (Takara). 293 T cells, which were serially transfected with pRK7-conl and pRK7-case, were washed in phosphate-buffered saline (PBS), and the supernatant was completely discarded. Next, two plates of cells were resuspended in 1X sodium dodecyl sulfate (SDS) buffer, followed by incubation at 99°C for 10 min. The scattered resuspension solution was collected. The proteins were separated on 10% SDS polyacrylamide gels at 80 V for 20 min and then at 130 V for 1 h. The proteins were semidry-blotted onto polyvinylidene fluoride membranes at 320 mA for 1 h. After blocking with 5% milk in PBS-Tween 20 (PBST) for 1 h, the membrane was incubated with mouse anti-FLAG monoclonal antibodies (Invitrogen) overnight at 4°C. The membrane was washed thrice with PBST for 30 min, incubated for 1 h with rabbit anti-mouse IgA/HRP (Invitrogen), and washed thrice for another 30 min. The membrane was incubated in enhanced chemiluminescence (ECL) Plus for 2 min, and the emitted light was detected using a CCD camera.

## Results

### Clinical observations

**Fig. 2:**
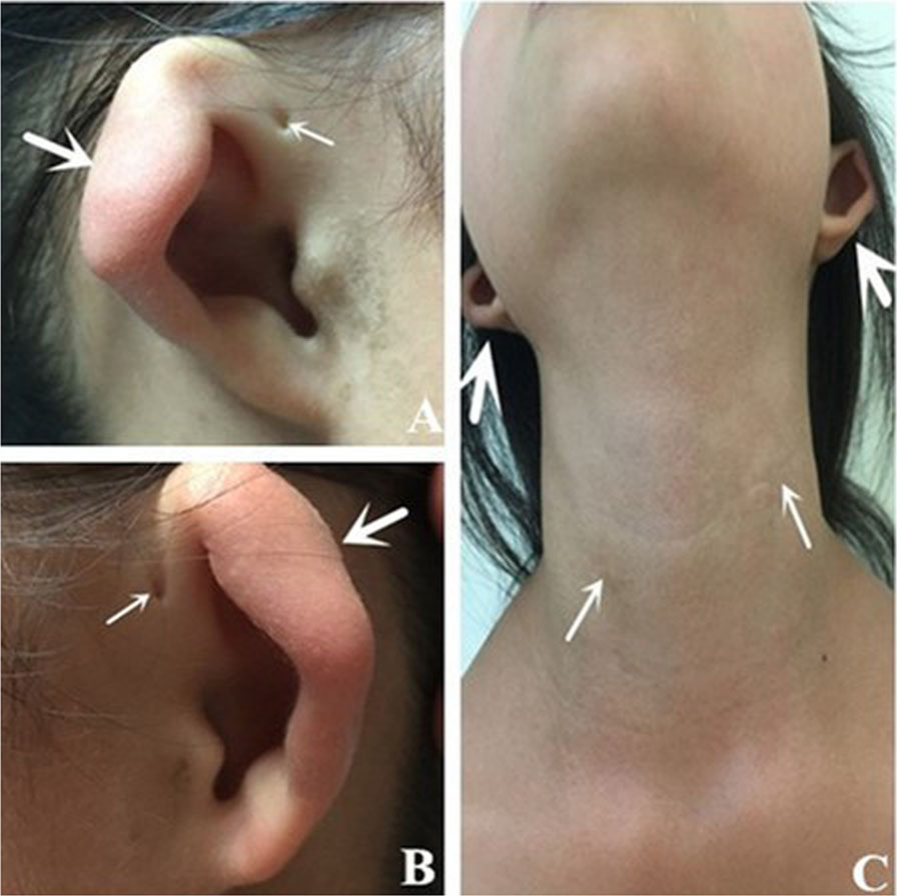
Images of both ears with microtia, preauricular pits, and branchial cleft fistulae in subject III:5. **a** and **b** Small arrows indicate preauricular pits, and large arrows indicate microtia. **c** Small arrows indicate branchial cleft fistulae, and large arrows indicate microtia

**Fig. 3:**
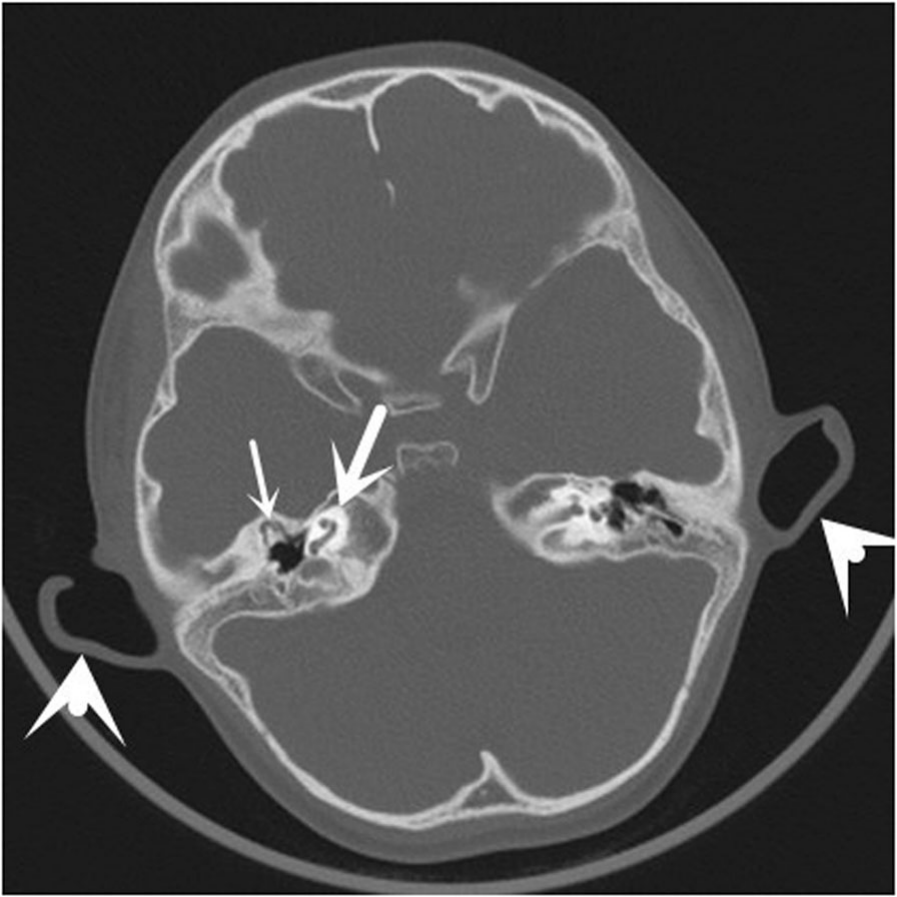
Axial computed tomographic images of the temporal bone from subject III:5. The small arrow indicates a malformed middle ear, including anteriorly displaced abnormal ossicles and hypoplastic mastoid cells. The middle arrow indicates malformed cochlea. The large arrow indicates microtia

As shown in Fig. 1, III:2 is the proband. Subject II:2 and her children (III:1, III:2) had bilateral preauricular pits. III:1 presented with bilateral branchial cleft fistulae and microtia (Fig. 2). Pure tone audiometry of III:1 showed bilateral conductive hearing loss. Bilateral middle ear malformation and inner ear hypoplasia were observed in the computed tomography (CT) scan (Fig. 3).

Subject III:2, the 5-year-old proposita, underwent a hearing test when she was 3 years old. Auditory brainstem response (ABR) testing revealed otitis media in both ears. The threshold of bone-conducted ABR was 20 dB nHL for both sides, while the threshold of air-conducted ABR was 55 dB nHL on the left side and 60 dB nHL on the right side. She had both microtia and facial hypoplasia on the right side. She also had palatoschisis and bilateral branchial cleft fistulae. Magnetic resonance imaging (MRI) showed bilateral otitis media and bilateral posterior semicircular canal and cochlear dysplasia. MRI also demonstrated thinner bilateral facial nerves than normal.

Subject II:6 had bilateral mixed hearing loss and preauricular pits. He had branchial cleft fistula only on the left side. Hydronephrosis and abnormal renal function had been detected several years ago. However, these symptoms were not present at the recent clinical evaluation. Renal ultrasonography showed bilateral mild diffuse echo change and slightly separated collecting system on the right side. CT showed bilateral inner ear hypoplasia and otitis media on the right side. His 3-year-old son (III:6) was negative for these findings by ABR, CT, and renal ultrasonography; however, his speech was not normal.

### Genetic observations

**Fig. 4:**
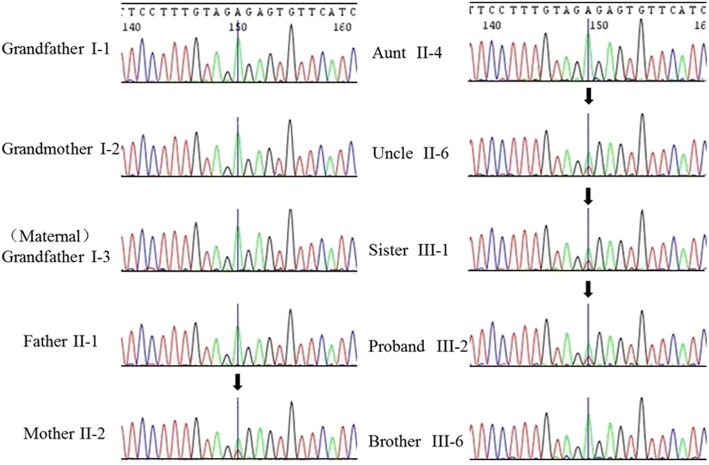
*EYA1* gene test in this family revealed a novel heterozygous nonsense variation (c.967A > T; p: Arg323X) in exon 11 in members with various physical anomalies (II:2, III:6, III:1, and III:2) (arrow). The normal family members harbored no mutations in *EYA1*

All 18 exons of *EYA1* and their flanking intron sequences were analyzed for all the living members in this family. Direct sequencing of the *EYA1* gene revealed that all the affected patients (II:2, III:6, III:1, and III:2) in this family carried a novel heterozygous nonsense variation (c.967A > T according to the GenBank transcript ID: NM_000503; p: Arg323X) in exon 11 (Fig. 4). The mutation was located at the beginning of exon 11 of *EYA1*. Meanwhile, none of the unaffected family members (I:1, I:2, I:3, II:1, II:4, or III:6) carried the mutation in *EYA1*. This mutation was not observed in any current database, including the HapMap, ExAC, and 1000 Genomes projects. Thus, the mutation is unlikely to represent genetic polymorphism.

### Minigene results

**Fig. 5:**
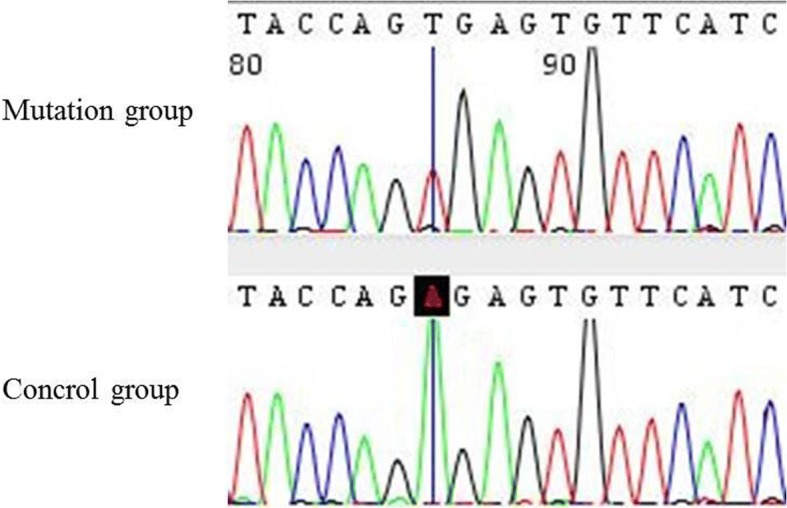
Results of the minigene experiment. Minigene experiments showed no change in RNA level according to the point change

Agarose gel electrophoresis results showed the wild-type and mutant gene to have a band of the same size (Additional file 2: Figure S1). cDNA sequence analysis confirmed the transcripts as wild-type, consistent with the reference sequence (Fig. 5). Although in silico analysis suggested the possibility of aberrant splicing being caused by c.967A > T, we did not detect any abnormal transcripts. The results showed that the mutation did not affect splicing, which caused a truncated protein to be the genetic cause of BOR syndrome in this family.

### Western blot analysis

**Fig. 6:**
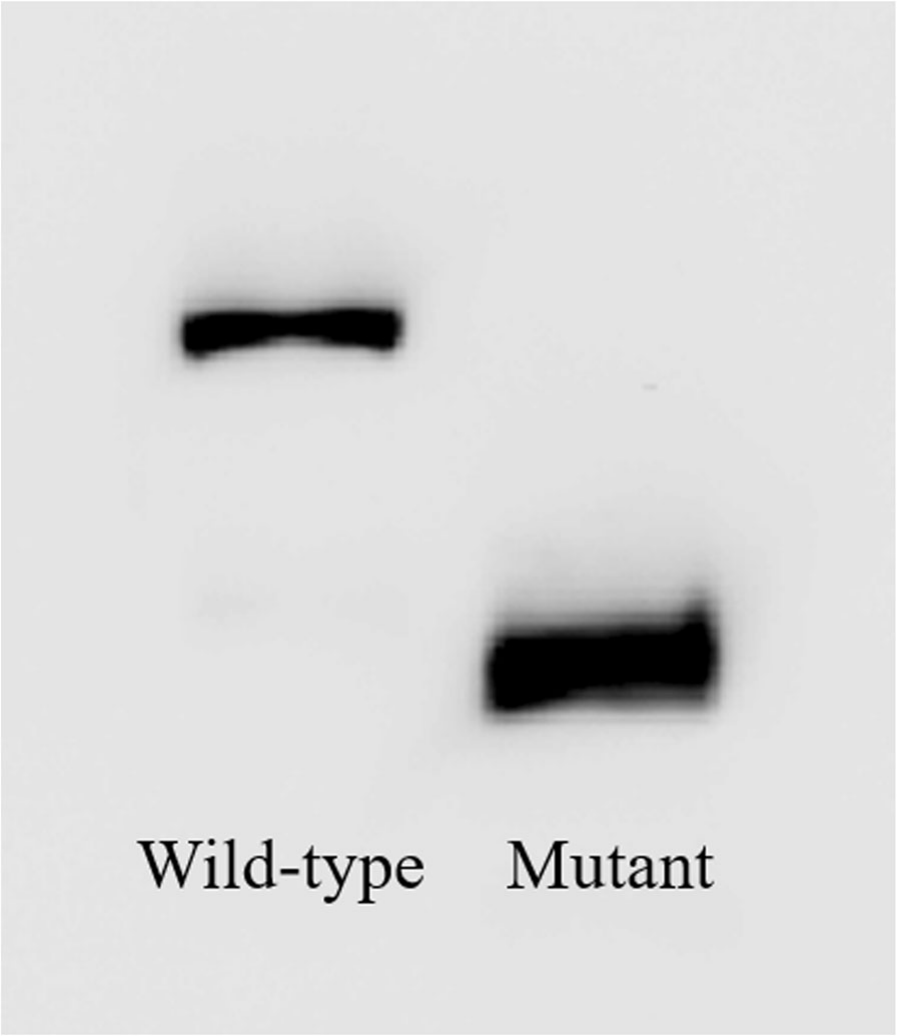
Western blotting shows that the protein encoded by the mutated plasmid was truncated compared to the wild type

Western blotting was performed to visualize the expressed proteins with a molecular weight of ~ 65 kDa for wild-type EYA1. The results showed that both wild-type and mutant proteins were expressed, but the mutant protein was significantly shorter, about half the size of the wild-type protein. This further proves that c.967A > T may lead to the premature termination of the protein (Fig. 6).

## Discussion

BOR syndrome is one of the most common autosomal dominant syndromes, characterized by five major phenotypes, namely, hearing loss (about 98%), preauricular pits (about 83%), branchial fistulae (about 68%), renal anomalies (about 38%), and external ear abnormalities (about 31.5%), based on studies on 40 families (Table 1) [2]. Some patients with branchial cleft, hearing loss, and preauricular pits have been found with branchio-otic (BO) syndrome [18], whereas others with hearing loss, branchial cleft, and obvious ureteral deformity are evaluated with branchio-oto-ureteral (BOU) syndrome [19]. It is well known that BO and BOU syndromes share the same pathogenic gene: *EYA1* [8, 20–22].

**Table 1:** Criteria for BOR syndrome

	Percentage (%)
Major Anomalies	
Hearing loss	98.5
Preauricular pits	83.6
Branchial anomalies	68.5
Renal anomalies	38.2
Minor Anomalies	
External ear anomalies	31.5
Middle ear anomalies	
Inner ear anomalies	
Other: facial asymmetry, palate abnormalities	

*EYA1* is a member of the *EYA* family, and other members include *EYA2*, *EYA3*, and *EYA4* [23]. The online website http://www.hgmd.cf.ac.uk/ac/index.php records 164 mutations related to BOR syndrome (last accessed, April 2016), of which 152 mutations are in *EYA1*, including missense, nonsense, and frameshift mutations [24–26].

The clinical phenotypes of BOR syndrome showed high heterogeneity [27]. Phenotypic variations are common for affected lineages because of the incomplete penetrance and variable expressivity within or between families. The affected members of this family also showed different phenotypes. For example, II:6 presented with all symptoms of BOR syndrome. III:1 and III:2 had preauricular pits, hearing loss, and branchial fistulae, whereas II:2 had only preauricular pits and hearing loss, indicating that II:2 strictly showed BO syndrome characteristics.

Genetic testing is increasingly becoming an important tool for clinical diagnosis. Genetic testing for this family revealed that all patients had a novel variation (c.967A > T, p: Arg323X) in exon 11 of *EYA1*. The variation produced a premature termination codon, which caused premature termination of translation. The online tool MutationTaster predicted that an alternative splicing site was located at the beginning of exon 11, which could possibly be altered by the mutation. Based on this hypothesis, minigene analysis was used to investigate whether this mutation affected splicing. However, no difference was observed between the mutant and the control. Hence, we speculated that this change produced only a termination codon.

Xu [28] reported an *Eya1*-knockout mouse model with hearing and renal defects and aberrant organ development. In addition, the morphogenesis of some parts, such as the thymus, parathyroid, and thyroid, was affected [29]. *EYA1* is a key gene for mammalian organogenesis, mutations in which result in multiple organ malformation. Similar to other EYA family members, EYA1 possesses a highly conservative 271-amino acid C-terminal EYA domain and a divergent N-terminal transactivation domain for protein–protein interactions [30]. Although EYA1 is a transcriptional activator, it did not bind directly to DNA in in vitro experiments. Co-factors of EYA1 are members of the SIX family of proteins. All SIX proteins possess two parts: one is the highly conserved SIX domain (SD) with DNA-binding ability and the other is an N-terminal domain that interacts with other proteins, including EYA. Several studies have shown that most of the SIX members and EYA1 are expressed during the development process. Interestingly, *SIX1*, *SIX5*, and *EYA1* are associated with BOR syndrome. Among these, *EYA1* mutations occur in approximately 41% of patients with BOS1 or BOR1 syndrome, and *SIX1* mutations affect 3.5~ 4.5% of patients with BOS3 or BOR3 syndrome, whereas *SIX5* has been mentioned in only one report. Furthermore, the association of the *SIX5* mutation with BOR syndrome has been recently disputed [31], as *Six*^*−/−*^ mice were found to develop only cataract. *SIX1* and *EYA1* are expressed in the growing basal plates, which include the ear placodes and the developing kidney [32], and they regulate the expression of other genes that are critical for the development of sense organs [33]. Loss of function of these two genes in other species causes placodal defects, alterations in the gene expression profile, cell apoptosis, reduction in cell proliferation in several placodes, etc. [28, 29].

**Fig. 7:**
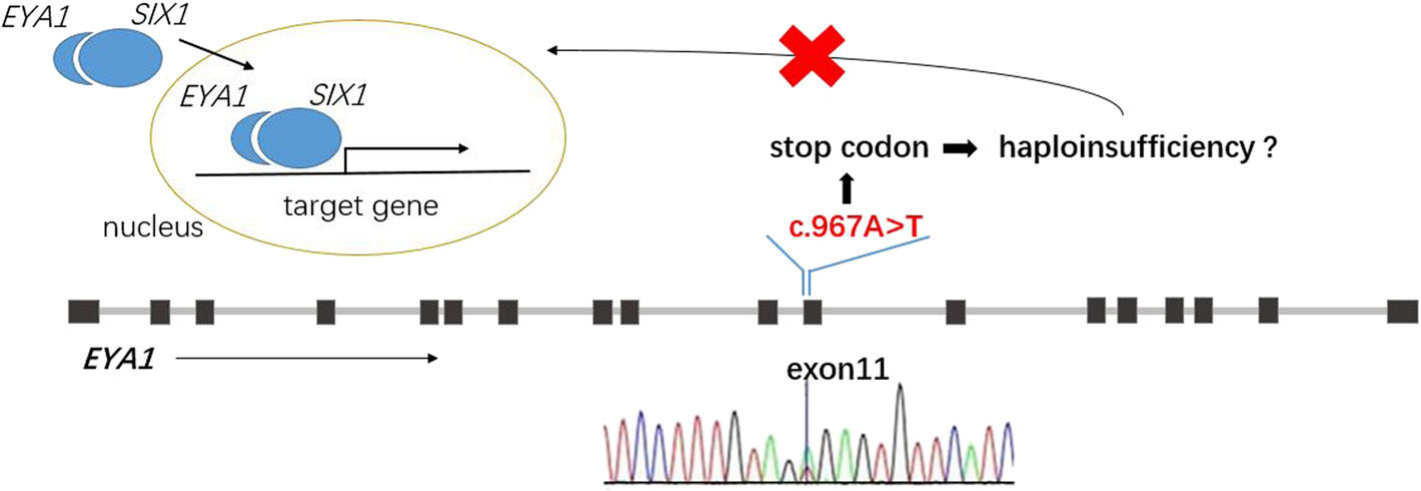
Mutation in *EYA1* exon 11 generates a stop codon. The haploinsufficiency of normal EYA1 disrupts SIX1 binding, which is necessary for the activation of target genes

Since the role of *EYA1* in development is crucial, it is related to four diseases as a pathogenic gene: otofaciocervical syndrome, anterior segment anomaly, BO syndrome, and BOR syndrome. Because the known pathogenic mutations of *EYA1* are loss-of-function mutations, haploinsufficiency might be the main pathological mechanism (Fig. 7).

Owing to the strong heterogeneity of clinical phenotypes, it is sometimes difficult for BOR syndrome to be diagnosed only based on clinical phenotypes, necessitating genetic testing for its early diagnosis. Early diagnosis would be beneficial for patients with BOR syndrome, as they could be sensitive to lifestyle changes and monitoring of kidney function later in life.

In this study, we identified a novel mutation in *EYA1*; however, we have not investigated the molecular mechanisms via which this mutation affects the pathogenesis and prognosis of the disease, which warrants further investigation. In addition, a limitation of this study is that the complete medical details of all the family members are not available.

## Conclusion

In summary, we report a novel nonsense mutation in *EYA1* as a causative mutation for BOR syndrome. We highlight that combining molecular tests with the analysis of clinical phenotypes would contribute to the timely diagnosis and treatment of BOR syndrome.

## Additional files

Additional file 1: **Table S1.** Primers used for gene analysis and DNA concentration. The table lists the primers we used in this experiment. F: forward primer; R: reversed primer. (DOCX 18 kb)

Additional file 2: **Figure S1.** Electrophoregram. In minigene search, after extracting RNA from cells with Trizol, we carried out the reverse-transcription (RT) and PCR amplification experiments. The electrophoregrams, SF1a shows RNA products and SF1b shows the PCR products, imply no difference between the mutant and wild-type. (ZIP 278 kb)

## Abbreviations

ABR: Auditory brainstem response
BO: Branchio-otic syndrome
BOR: Branchio-oto-renal syndrome
BOU: Branchio-oto-ureteral syndrome
CT: Computed tomography
MRI: Magnetic resonance imaging
PBS: Phosphate-buffered saline
PBST: PBS-Tween 20
PCR: Polymerase chain reaction
SDS: Sodium dodecyl sulfate buffer
