# Supplementary material for: A novel mutation in EYA1 in a Chinese family with Branchio-oto-renal syndrome
Source: BMC Med Genet. 2018 Aug 7;19:139. doi: 10.1186/s12881-018-0653-2 (PMC6081847; doi:10.1186/s12881-018-0653-2)
Supplement: Supplementary file 1 — Table S1. Primers used for gene analysis and DNA concentration. The table lists the primers we used in this experiment. F: forward primer; R: reversed primer. (DOCX 18 kb) [file 12881_2018_653_MOESM1_ESM.docx]

# Additional Table 1. Primers used for gene analysis and DNA concentration

| **Primer Name** | **Sequencing (5'--3')** | **Use** |
| --- | --- | --- |
| *EYA1*-1F | TGAGTCAGACAGAGCAGTGC | *EYA1* Exons PCR |
| *EYA1*-1R | TCGCTCATAATTTGGAGGTTGG | *EYA1* Exons PCR |
| *EYA1*-2F | TTCTCAGCCATGTGCTCTGT | *EYA1* Exons PCR |
| *EYA1*-2R | CAAGCACACACACACAAACTGT | *EYA1* Exons PCR |
| *EYA1*-3F | CCTTGTGACTTGATGAACTGTGT | *EYA1* Exons PCR |
| *EYA1*-3R | TGGAAACATGAAGCTCCTAACA | *EYA1* Exons PCR |
| *EYA1*-4F | AACTTGTGACAATAGATTGTTTCCT | *EYA1* Exons PCR |
| *EYA1*-4R | GCAACCACTTCTTCACAATGAAT | *EYA1* Exons PCR |
| *EYA1*-5F | TTGCGAAGATTGTGATGACG | *EYA1* Exons PCR |
| *EYA1*-5R | TCAGTGTCTCCACTACATGATGC | *EYA1* Exons PCR |
| *EYA1*-6F | GCATCATGTAGTGGAGACACTGA | *EYA1* Exons PCR |
| *EYA1*-6R | CCTCAAGATAAGGAGCTACCAGT | *EYA1* Exons PCR |
| *EYA1*-7F | GAAGGGATATGTCTTGAAGTGC | *EYA1* Exons PCR |
| *EYA1*-7R | CAATCCAGTTGCCATCATCA | *EYA1* Exons PCR |
| *EYA1*-8F | TGCAAATTGAAAAGTTCATTGTC | *EYA1* Exons PCR |
| *EYA1*-8R | CCAATCTGAAAACCAAACAACTC | *EYA1* Exons PCR |
| *EYA1*-9F | TTGTTTGCAAGTCTGAGCTCTT | *EYA1* Exons PCR |
| *EYA1*-9R | ATGCCACAGTCATGCTGCTT | *EYA1* Exons PCR |
| *EYA1*-10F | ACAACCTGTTGCCTCTCACC | *EYA1* Exons PCR |
| *EYA1*-10R | TGGCAATGATACAGATCAGC | *EYA1* Exons PCR |
| *EYA1*-11F | TTGCACCTCATCCAAAGTTAT | *EYA1* Exons PCR |
| *EYA1*-11R | TTAATTGAACAAAGTGTATCTTATTGC | *EYA1* Exons PCR |
| *EYA1*-12F | CCCAATGCCTGCTTCCTCTT | *EYA1* Exons PCR |
| *EYA1*-12R | ACCAACAAACCTCTGTCTCAC | *EYA1* Exons PCR |
| *EYA1*-13/14F | TCCTTAACGATGTTGCTTCTTTC | *EYA1* Exons PCR |
| *EYA1*-13/14R | GGCCAGTGAGATGAAACTGC | *EYA1* Exons PCR |
| *EYA1*-15/16F | AAGAGATGACTTCTGTAACCAAACC | *EYA1* Exons PCR |
| *EYA1*-15/16R | TGTTTCTCTTACGTCTAAATCCAGTT | *EYA1* Exons PCR |
| *EYA1*-17F | GTGCTGTGGCACATACAACC | *EYA1* Exons PCR |
| *EYA1*-17R | GAGTACTGCACATATTCATCACGTT | *EYA1* Exons PCR |
| *EYA1*-18F | GCCATATTTGGCAACGTTCT | *EYA1* Exons PCR |
| *EYA1*-18R | AGACGTGGTCCTCCATTCAC | *EYA1* Exons PCR |
| Mini-x/x-F | TGCTCTAGAGAAAACCTGGAATGT  TCTAAACTGAAAT | Minigene PCR |
| Mini-x/x-R | CCGCTCGAGGAAGCTAAATCCCCCAGACT | Minigene PCR |
| E-CDNA-F | GCTCTAGAATGGAAATGCAGGATCTAAC | CDNA PCR |
| E-CDNA-R | CGGAATTCTTACAGGTACTCCAGTTCCA | CDNA PCR |
| E-mut-F | GTGAGTGTTCATCTGGGACTTGGAT | point mutation |
| E-mut-R | TCAAGATCAGAATCTGGGGGAGGTG | point mutation |
|  |  |  |

The table lists the primers we used in this experiment. F: forward primer; R: reversed primer
