# Supplementary figures and images for: A novel mutation in EYA1 in a Chinese family with Branchio-oto-renal syndrome
Source: BMC Med Genet. 2018 Aug 7;19:139. doi: 10.1186/s12881-018-0653-2 (PMC6081847; doi:10.1186/s12881-018-0653-2)

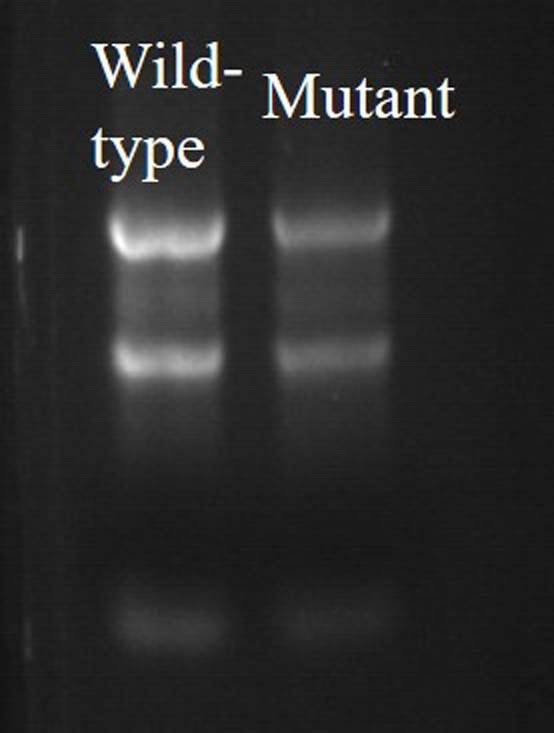

Supplement: Supplementary file 2 — Figure S1. Electrophoregram. In minigene search, after extracting RNA from cells with Trizol, we carried out the reverse-transcription (RT) and PCR amplification experiments. The electrophoregrams, SF1a shows RNA products and SF1b shows the PCR products, imply no difference between the mutant and wild-type. (ZIP 278 kb) [file 12881_2018_653_MOESM2_ESM.zip › AF1aR4.tif]
